# Tubal Pregnancy

**Published:** 1911-06

**Authors:** J. R. B. Branch

**Affiliations:** Attending Gynecologist to the Macon Hospital


### TUBAL PREGNANCY.

By J. R. B. Branch, M.D.

*Attending Gynecologist to the Macon Hospital*

### Frequency.

Up to 1883 when Tait performed the first operation for ruptured tubal pregnancy the condition was thought to be a rare one. Since that time, however, a marked increase in frequency has been noted, due, perhaps to more careful diagnosis, though to some extent to an increase in certain conditions favoring its occurrence. As in other cases the affection seems to run in waves. During the year 1909-1910 we saw between 40 and 50 cases in the gynecological clinic of the Johns Hopkins Hospital, more than had been observed in three previous years. One day we had two cases. During my twelve months in Macon I have seen or heard of only three cases.

### Etiology.

The etiology is obscure and real evidence to support any one view is quite insecure, for there is any number of theories. The most plausible ones may be divided into two classes (1.) Conditions which interfere with the mechanical passage of the ovum down the tube. (2.) Physical and developmental condition favorng decidual formation in the tubes.

### Situation.

The ovum may become implanted at any point in the Fallopian tube and so we have ampullar, isthmic and interstitial pregnancy. The ampullar being the commonest, interstitial being the rarest form.

We shall interest ourselves more in the clinical side of the subject and not enter into the mode of implantation, decidual formation, etc.

### Termination.

In a large majority of cases the pregnancy terminates in abortion or rupture, though, perhaps, the terms intra tubal and extra tubal rupture are more suitable. If the pregnancy be ampullar, intra tubal is the rule, if isthemic or interstitial, rupture into the peritoneal cavity is more common. As in uterine abortion we have a complete and incomplete, the latter being more common. The direct cause of rupture may be traumatic brought about by vaginal examination, coitus, straining at stool, or any other exertion, or more commonly it occurs spontaneously.

### Symptoms.

The symptoms of an unruptured tubal pregnancy are fairly characteristic. Let us give a typical history. A young woman of thirty comes in, complains of pain in the left lower abdominal quadrant. She is married, has one child six years old, no subsequent pregnancy. Menstrual history normal up to six weeks ago, her regular period failed to appear when due; two weeks later she began to have some pain in the left lower abdomen and some uterine bleeding which has persisted to the present time. She had some subjective signs of pregnancy. She has been curetted before coming in for the metrorrhagia with no relief. On pelvic examination the cervix is slightly softened, the uterus larger and softer than normal, there is a tender, velvety mass to the left of the fundus.

Should rupture have occurred one wud have perhaps the following addition to the history. Whlie attending to some house work she was seized with a sudden, sharp, agonizing pain in the left side, completely doubling her up, cuasing her to vomit. The pain may or may not have subsided spontaneously. In addition to the findings mentioned above, one might feel in the cul-de-sac a crepitant soft tumor absolutely characteristic of blood clots. Of course, where the hemorrhage has been severe the patient comes in faint, weak and pallid collapse with sub-normal temperature.

### Treatment.

Most operators agree now that early operation is indicated in unruptured cases preferably an abdominal incision with removal of the tube and its contents, for one never knows whether or not rupture with its consequent hemorrhage will occur. We need not go into detail regarding the technique of the operation, though we emphasize one point. The tube should be resected at the cornu, not merely cut off and ligated. I have seen an ectopic pregnancy develop in the stump of the patient's left tube which one year before had been removed with its ovary for ruptured tubal pregnancy. The patient recognized the condition herself the second time, and gave her diagnosis on admission. It is in the ruptured cases seen after severe hemorrhage has taken place that there is such a difference of opinion as to treatment Hunter Robb of Cleveland, has for several years been advocating delay where rupture has occurred and the patient is in shock. He bases his opinion upon some clinical experience and some very interesting experiments upon dogs where he demonstrates that hemorrhage per se is not the case of death but "shock" therefore, before operating and adding to shock, stimulate the patient until operation is safe. The majority of gynecologists advocate early operation with ligation of the bleeding points, and if patient's condition warrants it, removal of the blood and products of conception. The only case we lost out of the 40 odd referred to above was one in which operation was delayed 48 hours in a vain endeavor to combat shock. We do not claim to have added anything new to the subject treated, merely to have reviewed some features of it interesting to us. Free use has been made of the writing of Writridge, Williams, Howard A. Kelly and E. E. Montgomery, to whom due acknowledgement is given.

